# Lactate utilization in Lace1 knockout mice promotes browning of inguinal white adipose tissue

**DOI:** 10.1038/s12276-024-01324-w

**Published:** 2024-11-07

**Authors:** Youn Ju Kim, Sang Gyu Lee, Su In Jang, Won Kon Kim, Kyoung-Jin Oh, Kwang-Hee Bae, Hye Jin Kim, Je Kyung Seong

**Affiliations:** 1 Laboratory of Developmental Biology and Genomics, Research Institute for Veterinary Science, College of Veterinary Medicine, Seoul National University, Seoul, Republic of Korea; 2 Korea Model animal Priority Center (KMPC), Seoul National University, Seoul, Republic of Korea; 3 Division of Endocrine and Kidney Disease Research, Department of Chronic Disease Convergence Research, Korea National Institute of Health, Korea Disease Control and Prevention Agency, Cheongju, Republic of Korea; 4 Interdisciplinary Program in Bioinformatics, Seoul National University, Seoul, Republic of Korea; 5 Metabolic Regulation Research Center, Korea Research Institute of Bioscience and Biotechnology (KRIBB), Daejeon, Republic of Korea; 6 Department of Functional Genomics, KRIBB School of Bioscience, Korea University of Science and Technology (UST), Daejeon, Republic of Korea; 7 Interdisciplinary Program for Bioinformatics, Program for Cancer Biology, BIO-MAX/N-Bio Institute, Seoul National University, Seoul, Republic of Korea

**Keywords:** Fat metabolism, Homeostasis, Obesity, Metabolic syndrome, Metabolic syndrome

## Abstract

Recent studies have focused on identifying novel genes involved in the browning process of inguinal white adipose tissue (iWAT). In this context, we propose that the mitochondrial ATPase gene lactation elevated 1 (Lace1) utilizes lactate to regulate the browning capacity of iWAT, specifically in response to challenge with CL-316,243 (CL), a beta3-adrenergic receptor (β3-AR) agonist. The mice were injected with CL over a span of 3 days and exposed to cold temperatures (4–6 °C) for 1 week. The results revealed a significant increase in Lace1 expression levels during beige adipogenesis. Additionally, a strong positive correlation was observed between *Lace1* and *Ucp1* mRNA expression in iWAT under browning stimulation. To further explore this phenomenon, we subjected engineered Lace1 KO mice to CL and cold challenges to validate their browning potential. Surprisingly, Lace1 KO mice presented increased oxygen consumption and heat generation upon CL challenge and cold exposure, along with increased expression of genes related to brown adipogenesis. Notably, deletion of Lace1 led to increased lactate uptake and browning in iWAT under CL challenge compared with those of the controls. These unique phenomena stem from increased lactate release due to the inactivation of pyruvate dehydrogenase (PDH) in the hearts of Lace1 KO mice.

## Lace1 gene enhances browning capacity in adipose tissue

In recent years, researchers have found that certain conditions can change white fat cells into beige fat cells, a process called “browning.” In this study, scientists focused on finding new genes that help in the browning of white fat cells. They did this by treating mice with a compound that starts the browning process and studying changes in gene activity. They found that the Lace1 gene is very active in brown fat tissue and its activity increases during the browning of white fat. The main discovery of this research is that not having the Lace1 gene leads to browning of white fat tissue in mice, suggesting that Lace1 may slow down this process. The researchers conclude targeting the Lace1 gene could be a new way to promote the browning of white fat and fight obesity. This summary was initially drafted using artificial intelligence, then revised and fact-checked by the author.

## Introduction

Stimuli such as cold exposure, treatment with β3-adrenergic receptor (β3-AR) agonizts such as CL-316,243 (CL), and exercise can induce the conversion of inguinal white adipose tissue (iWAT) into beige adipose tissue^1^. This phenomenon is referred to as the “browning” of white adipocytes^2^. Beige adipocytes are similar to brown adipocytes and present characteristics such as multilocular lipid droplets and abundant mitochondria. Their primary role involves the dissipation of energy to generate heat. Therefore, the browning of iWAT is recognized as an important mechanism in obesity research.

CL, an agonist of β3-AR, stimulates β3-AR by binding to its G protein α-subunit. This activation initiates the protein kinase A (PKA) pathway within adipocytes by triggering adenylate cyclase and cyclic AMP (cAMP) production^3^. Consequently, this process induces lipolysis, which converts triacylglycerol (TG) into fatty acids (FAs) via the phosphorylation of hormone-sensitive lipase (HSL) in adipocytes. The generated free FAs then activate the induction of uncoupling protein 1 (Ucp1) in mitochondria, which is the thermogenic pathway^4^. UCP1 is a mitochondrial protein and a key gene that is upregulated during the browning process^5^. Recent studies have revealed novel forms of Ucp1-independent thermogenesis, but these processes have been poorly explored^6–8^. These current research trends have ignited the quest to identify new genes involved in the browning process.

Hence, we conducted an RNA-seq analysis of iWAT following CL treatment. The aim of this study was to assess the genes whose expression is upregulated in beige adipocytes compared with white adipocytes. From the pool of 292 genes whose expression was upregulated in the CL-injected group, 97 mitochondrial genes were selected for further investigation. Within this subset, we identified 11 genes that were enriched in BAT, leveraging the BioGPS database. Among these candidates, we chose to focus on lactation elevated 1 (Lace1), a gene with an as-yet-undetermined function and role, for our study.

Lace1 is a human homolog of the yeast mitochondrial ATPase family gene 1 (Afg1), a member of the SEC18-NSF, PAS1, CDC48-VCP, and TBP families^9^. ATPases are categorized into F-ATPase, P-ATPase, and V-ATPase, of which Lace1 is classified as an F_0_/F_1_ ATPase^10^. F_0_/F_1_ ATP synthase is an enzyme localized in the inner membrane of mitochondria, where it catalyzes the synthesis of ATP from ADP^11^. Recent studies have reported that ATPases regulate thermogenesis in a Ucp1-dependent or Ucp1-independent manner^12,13^. Lace1 is also a mitochondrial integral membrane protein that controls mitochondrial protein homeostasis^9^. Cesnekova et al. reported that Lace1 mediates the degradation of the nuclear-encoded complex IV subunits cytochrome c oxidase 4 (COX4), COX5A, and COX6A and is required for the normal activity of complexes III and IV of the respiratory chain^9^.

Consequently, Lace1, identified as a mitochondrial gene, has increased expression due to CL or cold exposure. These observations strongly suggest that Lace1 might serve as a potential novel marker for browning. These findings prompted us to further explore the role of Lace1 in white fat browning and ascertain its importance. Our approach involved the generation of Lace1 knockout (KO) mice. Our findings revealed that the Lace1 gene was upregulated during the browning process of iWAT. Strikingly, Lace1 KO mice displayed an increased capacity for browning. This intriguing outcome prompted us to meticulously document and analyze the intricate mechanisms behind this phenomenon. In this study, we investigated the role of Lace1 in white adipose tissue browning.

## Materials and methods

### Animals and experimental design

All animal experiments were performed according to the “Guide for Animal Experiments” (edited by the Korean Academy of Medical Sciences). These animal experiments were approved by the Institutional Animal Care and Use Committee of Seoul National University, Seoul, Korea (permission number: SNU-210217-9). Lace1 KO mice were used to delete exon 2 of the Lace1 gene via the CRISPR-Cas9 system by Macrogen (Korea). All the animals were housed under a 12-h light/dark cycle at 22 ± 1 °C and 50–60% humidity with a 12 h light/dark cycle in a specific pathogen-free facility.

### Physiological measurement

The fat mass and lean mass of the mice were measured via nuclear magnetic resonance (NMR) methods (Minispec LF-50, Bruker, Germany). The rectal temperature of the mice was recorded via a BAT-12 microprobe thermometer (Physitemp, USA).

### Protein extraction and western blotting

For protein expression analysis, all proteins were extracted via RIPA buffer (BR002, Biosolution, Korea) supplemented with protease inhibitor cocktail and phosphatase inhibitor cocktail (P3100-001, P3200-001, GeneDEPOT, USA). Equal amounts of proteins were separated on SDS‒PAGE gels and transferred to PVDF membranes. Primary antibodies targeting the following proteins were used: LACE1 (NBP1-89215, Novus Biologicals, CO, USA), UCP1 (ab10983, Abcam), MCT1 (ab250131, Abcam), MCT4 (ab74109, Abcam), LDHA (2012, CST), phospho-PDH (31866, CST), PDH (3205, CST), α-actin (A2066, Sigma‒Aldrich), and β-tubulin (ab2146, Abcam).

### RNA extraction and quantitative real-time PCR

For mRNA expression analysis, total RNA was extracted via TRIzol reagent (Invitrogen), and 1 µg of RNA was synthesized into cDNA via RT premix (K-2044-B, Bioneer, Korea). qPCR was performed via the SYBR Green Kit (BIO-92005, Meridian Bioscience, OH, USA) according to the manufacturer’s instructions. The fold change for all the samples was calculated via the 2^−ΔΔCt^ method. The primer sequences are listed in Table 1.

**Table 1:** Primers used for qRT-PCR analysis.

Target gene		Sequence (5′ → 3′)
*Lace1*	Forward	CCGAGGAAATCAGTCAAGAA (20 mer)
*Lace1*	Reverse	GGTGGTCTGTTGGATGTT (18 mer)
*Ucp1*	Forward	ACTGCCACACCTCCAGTCATT (21 mer)
*Ucp1*	Reverse	CTTTGGCTCACTCAGGATTGG (21 mer)
*Cidea*	Forward	ATCACAACTGGCCTGGTTACG (21 mer)
*Cidea*	Reverse	TACTACCCGGTGTCCATTTCT (21 mer)
*Elovl3*	Forward	TTCTCACGCGGGTTAAAAATGG (22 mer)
*Elovl3*	Reverse	GAGCAACAGATAGACGACCAC (21 mer)
*Cox8b*	Forward	GAACCATGAAGCCAACGACT (20 mer)
*Cox8b*	Reverse	GCGAAGTTCACAGTGGTTCC (20 mer)
*Cox7a1*	Forward	CAGCGTCATGGTCAGTCTGT (20 mer)
*Cox7a1*	Reverse	AGAAAACCGTGTGGCAGAGA (20 mer)
*Dio2*	Forward	CAGTGTGGTGCACGTCTCCAATC (23 mer)
*Dio2*	Reverse	TGAACCAAAGTTGACCACCAG (21 mer)
*Ldha*	Forward	TATCTTAATGAAGGACTTGGCGGATGAG (28 mer)
*Ldha*	Reverse	GGAGTTCGCAGTTACACAGTAGTC (24 mer)
*Ldhb*	Forward	TTGTGGCCGATAAAGATTACTCTGTGAC (28 mer)
*Ldhb*	Reverse	AGGAATGATGAACTTGAACACGTTGAC (27 mer)
*Mct1*	Forward	CATTGGTGTTATTGGAGGTC (20 mer)
*Mct1*	Reverse	GAAAGCCTGATTAAGTGGAG (20 mer)
*Mct4*	Forward	TCAATCATGGTGCTGGGACT (20 mer)
*Mct4*	Reverse	TGTCAGGTCAGTGAAGCCAT (20 mer)
*Hcar1*	Forward	GCTTACCCCTTCGGACAGAC (20 mer)
*Hcar1*	Reverse	ATGCTCCCGGCCCTATTCA (19 mer)

### Histopathology

All the tissues were fixed with 4% paraformaldehyde (HP2031, Biosesang) at room temperature overnight, embedded in paraffin, and sectioned into 3–5 μm sections. For H&E staining, all the slides were stained following the manufacturer’s instructions. For immunohistochemical staining, the slides were stained with an anti-UCP1 antibody (ab10983, Abcam).

### Indirect calorimetry

VO_2_ and energy expenditure were estimated by indirect calorimetry during CL challenge for 3 days and cold exposure (4 °C) for 7 days. During indirect calorimetry, all the mice were housed in a single cage with free access to food and water under a 12 h light/dark cycle. All the mice were monitored by PhenoMaster 7.5.6 (TSE system, Germany) during the measurement of VO_2_ and energy expenditure.

### L-Lactate measurement

Serum L-lactate levels were measured with an L-lactate assay kit (ab65331, Abcam, UK). All protocols were performed following the manufacturer’s instructions.

### Analysis of bulk-RNA sequencing and bioinformatics data

lllumina’s TruSeq Stranded mRNA LT Sample Prep Kit was used to prepare RNA-sequencing libraries and high-throughput sequencing was performed with Illumina’s NovaSeq 6000 Platform for each sample. The sequenced reads were mapped to the GRCm39 mouse reference genome via STAR v2.7.4a. Differential expression analysis was performed via the R packages DESeq2 v.1.32.0 and apeglm v.1.14.0 via the shrinkage method. Differentially expressed genes were identified with cutoffs of adjusted *P* values < 0.01 and log2-fold changes > 1 in the CL-challenged experimental group and adjusted *P* values < 0.05 and log2-fold changes > 0.58 in the Lace1 KO experimental group. All graphical visualizations were implemented in R via ggplot2 v3.3.5

### Brown adipocyte culture

When the brown preadipocyte cells reached 90–100% confluency, the cells were induced with induction medium (DMEM with 10% FBS, 0.5 mM isobutylmethylxanthine (I7018, Sigma, USA), 0.5 μM dexamethasone (D1756, Sigma), 125 μM indomethacin (I7378, Sigma), 1 nM T3 (T2877, Sigma) and 20 nM insulin (sc-360248, Santa Cruz)). After induction for 2 days, the medium was changed to insulin medium (DMEM with 10% FBS, 1 nM T3 and 20 nM insulin). Five days after differentiation, brown adipocytes were transfected with 50 nM Lace1 siRNA (30 nM) or negative control siRNA for 36 h via Lipofectamine RNAiMAX reagent (13778075, Thermo Scientific, USA) to generate Lace1-knockdown adipocytes. The predesigned siRNA directed against murine Lace1 mRNA (accession no. NM_001359297.1) was purchased from Bioneer (Daejeon, Korea).

### Beige adipocyte culture

Seven-week-old male C57BL/6N mice were sacrificed, and the stromal vascular fraction (SVF) was harvested from iWAT. The tissues were digested with 1.5 U/ml collagenase D (11088882001, Roche) in a 37 °C shaker for 30 min. The SVF pellets were incubated in collagen-coated plates with maintenance medium (DMEM/F12 with 10% FBS and 1X P/S) for 1 hr. After that, the medium was changed to fresh medium for removing immune cells, etc. Beige adipogenesis of preadipocytes of iWAT was induced according to a previous protocol^14,15^.

### Mitochondrial isolation

Mitochondrial and cytosolic isolation from differentiated brown adipocytes was performed with a mitochondrial isolation kit (ab110170, Abcam). All protocols were performed following the manufacturer’s instructions.

### ATP assay

ATP levels in fully differentiated brown adipocytes were analyzed via an ATP assay kit (K354-100, BioVision, CA, USA). All protocols were performed following the manufacturer’s instructions.

### Oxygen consumption ratio (OCR)

The oxygen consumption rate (OCR) of differentiated brown adipocytes from the control siRNA and *siLace1* knockdown (KD) groups was measured via a Seahorse Fe Extracellular Flux Analyzer (Agilent Technologies, CA, USA). For the measurement of the OCR, differentiated brown adipocytes were incubated at 37 °C in a non-CO_2_ incubator for 1 h with Seahorse XF DMEM (103575–100, Agilent) supplemented with 1 mM pyruvate (103577–100, Agilent), 2 mM glutamine (103579–100, Agilent), and 10 mM glucose (103578–100, Agilent). After incubation, the brown adipocytes were treated with 1.5 μM oligomycin, 2.0 μM carbonyl cyanide-4 (trifluoromethoxy) phenylhydrazone (FCCP), and 0.5 µM rotenone and antimycin (Rot/AA). All reagents used were from the Agilent Seahorse XF Cell Mito Stress Test Kit (103015-100, Agilent). The OCR of brown adipocytes was normalized to the protein amount.

### Statistical analysis

All the statistical data were analyzed via GraphPad Prism 7.0 (GraphPad Software, La Jolla, CA, USA). All the data are presented as the means ± standard errors of the means (SEMs). Statistical significance was analyzed by an unpaired *t-*test. Statistical significance was determined at *p < 0.05, **p < 0.01 and ***p < 0.001.

## Results

### Lace1 is a mitochondrial ATPase that is highly expressed in BAT

**Fig. 1:**
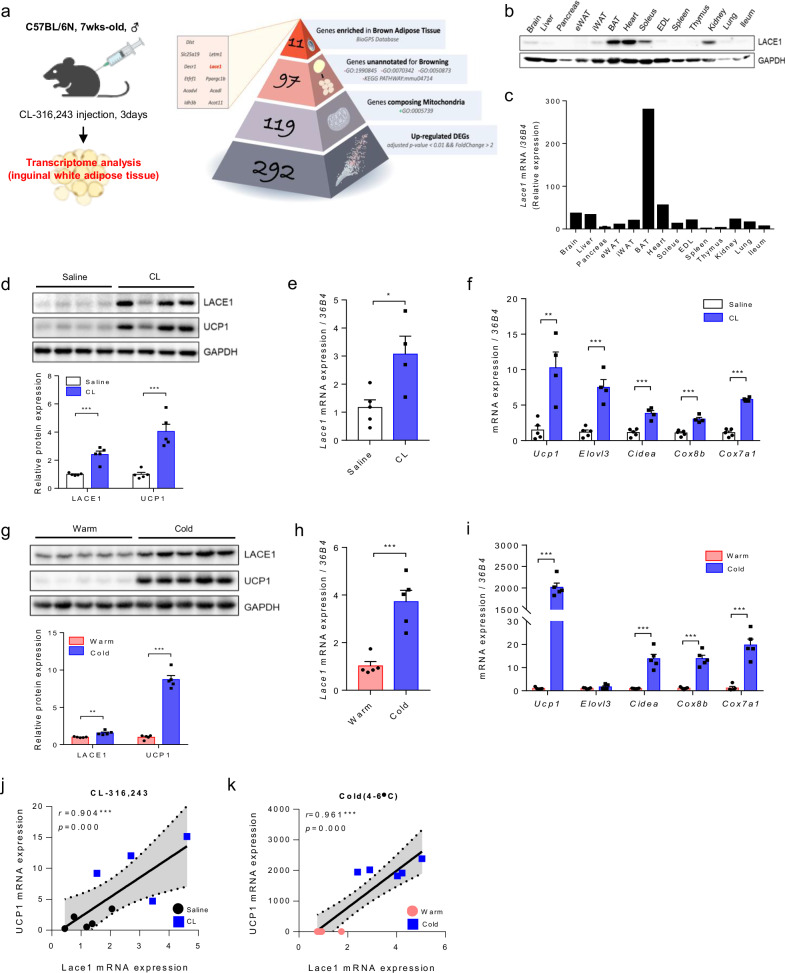
Lace1 is increased in iWAT by browning stimulation. **a** Transcriptomic analysis of genes upregulated in iWAT from 7-week-old male C57BL/6N mice subjected to CL challenge. **b** LACE1 protein expression in whole tissue of 7-week-old male C57BL/6N mice, *n* = 4. **c** *Lace1* mRNA expression in whole tissue of 7-week-old male C57BL/6N mice, *n* = 4. **d** LACE1 and UCP1 protein expression in iWAT upon CL challenge; *n* = 5 for all groups. **e** *Lace1* mRNA expression in iWAT upon CL challenge. Saline; *n* = 5, CL; *n* = 4. **f** Thermogenesis-related gene expression in iWAT upon CL challenge. Saline; *n* = 5, CL; *n* = 4. **g** LACE1 and UCP1 protein expression in iWAT upon cold exposure; n = 5 for all groups. **h** *Lace1* mRNA expression in iWAT upon cold exposure; *n* = 5 for all groups. **i** Thermogenesis-related gene expression in iWAT upon cold exposure; *n* = 5 for all groups. **j** Correlation between *Lace1* and *Ucp1* mRNA levels under CL challenge. Saline; *n* = 5, CL; *n* = 4. **k** Correlation between *Lace1* and *Ucp1* mRNA levels upon cold exposure; *n* = 5 for all groups. All experiments were performed after intervention. The values are presented as the means ± SEMs. Significance was calculated via an unpaired two-tailed Student’s *t*-test. *p < 0.05, **p < 0.01, ***p < 0.001.

To investigate novel genes involved in beige adipogenesis, we compared the CL-treated and untreated mice via the bulk RNA-seq technique. Among the 292 genes upregulated by CL challenge (adjusted *P* value < 0.01 and a fold change > 2 as the cutoff), 97 genes were identified as nonbrowning mitochondrial genes; these genes were annotated as mitochondrial genes (GO:0005739) and unannotated for browning-related biological processes (GO:1990845, GO:0070342, GO:0050873, KEGG PATHWAY: mmu04714). Using the BioGPS database to identify candidate genes of interest, we selected 11 genes highly expressed in BAT. In this research, we present Lace1, a highly enriched nonbrowning mitochondrial-annotated gene in BAT, as a novel gene involved in beige adipogenesis (Fig. 1a and Supplementary Fig. 3).

To clarify the involvement of this novel gene, Lace1, in beige adipogenesis, we first measured the expression level of Lace1 in the whole tissues of 7-week-old C57BL/6N male mice. The expression of LACE1 protein was highest in BAT, followed by the heart, soleus, and kidney (Fig. 1b). Compared with that in other tissues, *Lace1* mRNA was highly expressed in BAT (Fig. 1c). Next, as Lace1 expression is highest in BAT, we measured Lace1 expression during brown adipogenesis in immortalized brown preadipocytes. As a result, LACE1 protein levels were found to be increased during brown adipogenesis, similar to those of Ucp1 and peroxisome proliferator-activated receptor gamma coactivator 1-alpha (Pgc1α) (Supplementary Fig. 1a). *Lace1* mRNA was also increased during brown adipogenesis in a *Ucp1-*, cell death-inducing DNA fragmentation factor alpha-like effector A *(Cidea)*- and *Pgc1α*-dependent manner (Supplementary Fig. 1b–e). During beige adipogenesis, LACE1 protein levels, similar to those of UCP1, increased (Supplementary Fig. 1f). Additionally, *Lace1* mRNA expression increased during beige adipogenesis in a *Ucp1* mRNA expression-dependent manner (Supplementary Fig. 1g, h). These data suggest that Lace1 is highly expressed in the BAT of mice and that its expression increases during brown adipogenesis and beige adipogenesis.

To determine the localization of Lace1 expression, we confirmed the expression of Lace1 in the cytosol and mitochondria of preadipocytes and fully differentiated brown adipocytes via immortalized brown preadipocytes^15^. As expected, the LACE1 protein was expressed only in the mitochondria of fully differentiated brown adipocytes (Supplementary Fig. 2a). Consistent with these findings, we transfected fully differentiated brown adipocytes with siRNAs targeting the Lace1 gene. After transfection with *siLace1*, we attempted to confirm mitochondrial function in fully differentiated brown adipocytes by directly measuring the oxygen consumption rate (OCR). We found that the OCR of the *Lace1*-knockdown (KD) group of brown adipocytes was lower than that of the control siRNA group (Supplementary Fig. 2b–f). In particular, basal respiration and maximal respiration were lower in the Lace1-KD brown adipocytes than in the WT brown adipocytes. Lace1 is also known as a mitochondrial ATPase, which synthesizes ATP^9,16^. As with previous studies, we confirmed that ATP levels were lower in the *Lace1*-KD brown adipocytes than in the WT brown adipocytes (Supplementary Fig. 2g).

### Lace1 is increased in iWAT following CL challenge and cold exposure

Our transcriptomic data revealed that the *Lace1* gene is one of the genes upregulated in iWAT by CL challenge. Therefore, we hypothesized that Lace1 is increased in beige fat in a Ucp1 expression-dependent manner and is upregulated during beige adipogenesis. As expected, we confirmed that LACE1 protein levels, similar to those of Ucp1, are increased in CL-induced beige fat (Fig. 1d). Additionally, *Lace1* mRNA levels increased, similar to those of thermogenic genes (Fig. 1e and f). Next, we investigated Lace1 expression under cold exposure. Cold exposure induces browning of iWAT via the release of NE and β3-adrenergic stimulation^17,18^. Consequently, LACE1 protein levels were greater in cold-induced beige fat than in white fat (Fig. 1g). Similarly, *Lace1* mRNA expression increased, similar to that of thermogenic genes (Fig. 1h and i). We found that Lace1 was increased in a Ucp1-dependent manner in response to CL challenge and cold exposure; therefore, we performed a correlation analysis between *Lace1* and *Ucp1* mRNA expression. Interestingly, *Lace1* and *Ucp1* mRNA levels were positively correlated under CL challenge and cold exposure (CL challenge: *r* = 0.904, *p* = 0.000/cold exposure: *r* = 0.961, *p* = 0.000) (Fig. 1j and k). These data suggested that Lace1 is increased in iWAT following CL challenge and cold exposure.

### Lace1 deficiency increases browning of iWAT following CL injection

**Fig. 2:**
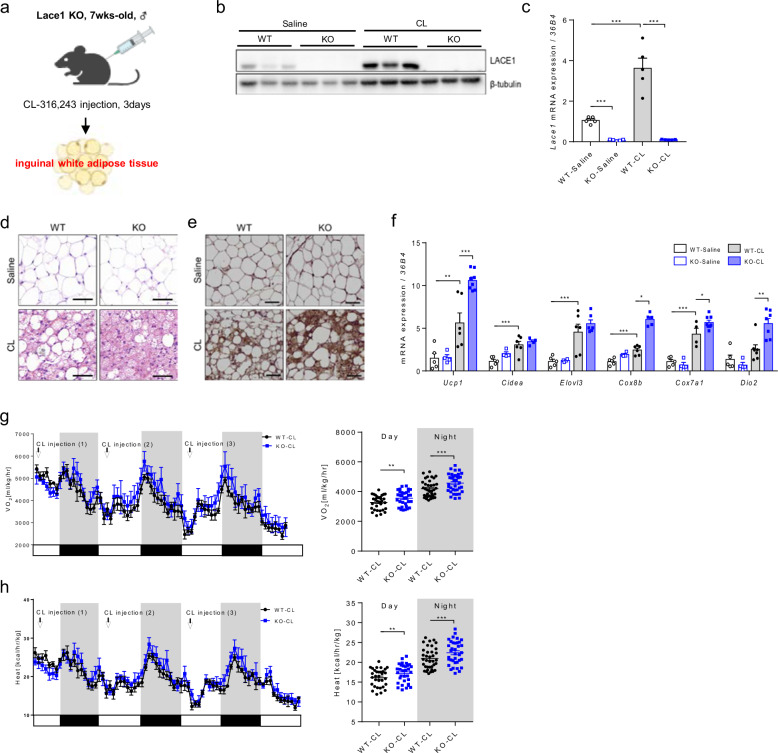
Lace1 deficiency increases browning of iWAT following CL injection. **a** Scheme of the CL challenge once a day for 3 days. **b** LACE1 protein expression in iWAT of the Lace1 KO mice upon CL challenge; *n* = 3 for all groups. **c** *Lace1* mRNA expression in iWAT of the Lace1 KO mice upon CL challenge. WT-saline; *n* = 5, KO-saline; *n* = 5, WT-CL; *n* = 5, KO-CL; *n* = 5. **d** Representative H&E staining of iWAT from the Lace1 KO mice upon CL challenge (scale bar = 50 μm); *n* = 3 per group. **e** UCP1 immunohistochemical staining of iWAT from the Lace1 KO mice upon CL challenge (scale bar = 50 μm); *n* = 3 per group. **f** Thermogenesis-related gene expression in iWAT of the Lace1 KO mice upon CL challenge. WT-saline; *n* = 5, KO-saline; *n* = 4, WT-CL; *n* = 6, KO-CL; *n* = 6–8. **g**, **h** Whole-body oxygen consumption rate (VO_2_) and heat generation in the Lace1 KO mice during CL challenge for 3 days; *n* = 5 for all groups. All experiments were performed after intervention. The values are presented as the means ± SEMs. Significance was calculated via an unpaired two-tailed Student’s *t-*test. *p < 0.05, **p < 0.01, ***p < 0.001.

To investigate the role of Lace1 in beige fat, we generated Lace1 null knockout (KO) mice via the CRISPR-CAS9 system. A scheme of the construction of Lace1 KO mice is presented in Supplementary Fig. 4. We administered CL to Lace1 KO mice according to this experimental design (Fig. 2a). First, we confirmed that *Lace1* protein and mRNA expression is downregulated in the iWAT of the Lace1 KO mice compared with the WT mice despite CL injection (Figs. 2b and c). Next, we investigated the browning capacity of iWAT in the Lace1 KO mice under CL challenge. Surprisingly, histological analysis revealed that the Lace1 KO mice had a greater beige fat content than the WT mice when challenged with CL (Fig. 2d). Together, the results of the UCP1 immunostaining analysis revealed that the Lace1 KO mice had more Ucp1-positive adipocytes than the WT mice did under CL challenge (Fig. 2e). Additionally, thermogenic marker gene levels were greater in the CL-induced beige fat of the Lace1 KO mice than in that of their control littermates (Fig. 2f). Ucp1 is critical for maintaining core temperature and increasing energy expenditure^19^. CL challenge and cold exposure-dependent oxygen consumption rate (VO_2_) are known to be derived from Ucp1 activity^20^. To explore the systemic metabolic effects of heightened browning in white adipose tissue of Lace1 KO mice, we conducted indirect calorimetry during the CL challenge. The Lace1 KO mice exhibited increased oxygen consumption (Fig. 2g) and heat generation after CL challenge (Fig. 2h). We hypothesized that the browning capacity of iWAT is decreased by Lace1 gene deletion compared with that of WT iWAT because Lace1 is upregulated in iWAT under browning stimuli such as Ucp1; however, surprisingly, we found that Lace1 deficiency increases the browning capacity of iWAT under CL challenge. On the basis of the above results, we explored whether this phenotype occurred in the Lace1 KO mice upon exposure to another stimulus, cold, as with the CL challenge.

### Lace1 deficiency results in increased browning of iWAT following cold exposure

**Fig. 3:**
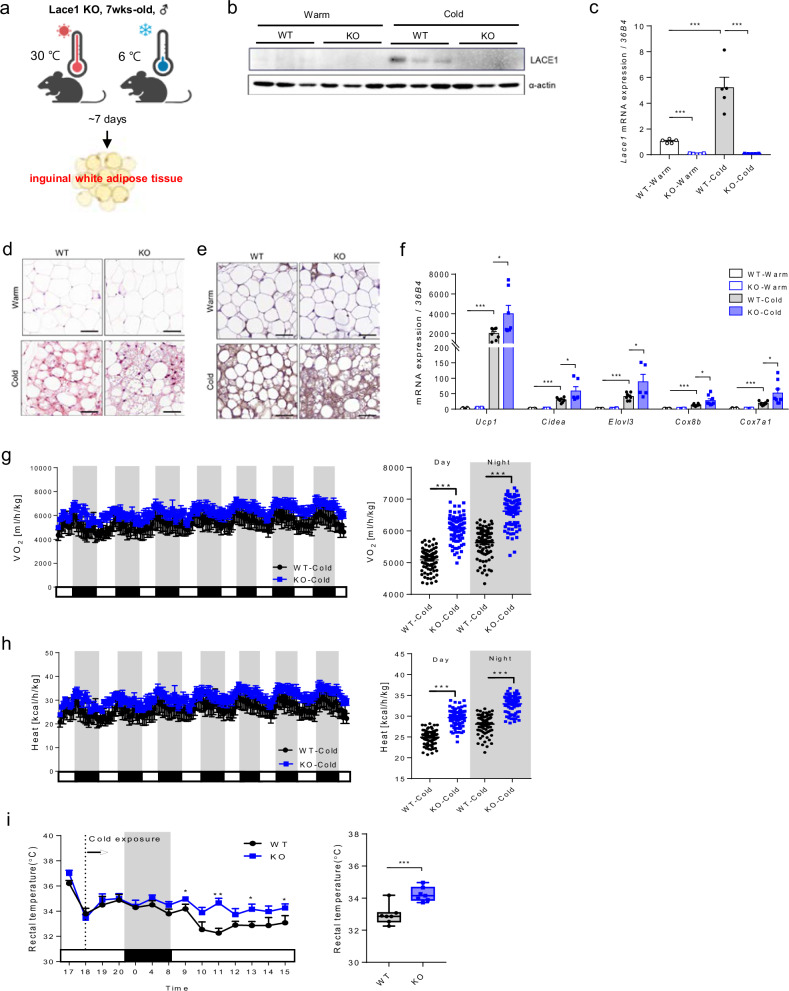
Lace1 deficiency increases browning of iWAT following cold exposure. **a** Scheme of cold exposure for 7 days. **b** LACE1 protein expression in iWAT of the Lace1 KO mice upon cold exposure; *n* = 3 for all groups. **c** *Lace1* mRNA expression in iWAT of the Lace1 KO mice upon cold exposure; *n* = 5 for all groups. **d** Representative H&E staining of iWAT from the Lace1 KO mice upon cold exposure (scale bar = 50 μm); *n* = 3 for all groups. **e** UCP1 immunohistochemical staining of iWAT from the Lace1 KO mice upon cold exposure (scale bar = 50 μm); *n* = 3 for all groups. **f** Thermogenesis-related gene expression in iWAT of the Lace1 KO mice upon cold exposure; *n* = 7 for all groups. **g**, **h** Whole-body oxygen consumption rate (VO_2_) and heat generation in the Lace1 KO mice during cold exposure for 7 days; *n* = 5 for all groups. **i** Rectal temperature during cold exposure in the Lace1 KO mice; *n* = 8 for all groups. All experiments were performed after intervention. The values are presented as the means ± SEMs. Significance was calculated via an unpaired two-tailed Student’s *t*-test. *p < 0.05, **p < 0.01, ***p < 0.001.

For analysis of the phenotype of Lace1 KO mice in response to cold stimuli, the mice were exposed to cold at 4–6 °C for 7 days (Fig. 3a). As shown in Fig. 3b and c, *Lace1* protein and mRNA expression was downregulated in the iWAT of the Lace1 KO mice compared with that of the WT mice despite cold exposure. Histological analysis revealed that the Lace1 KO mice had greater beige fat content than the WT mice when challenged with cold (Fig. 3d). Additionally, Ucp1 immunostaining analysis revealed that the iWAT of the Lace1 KO mice had more Ucp1-positive adipocytes than did that of the WT mice upon cold exposure (Fig. 3e). Additionally, thermogenesis-related gene expression was greater in the iWAT of the Lace1 KO mice than in that of the WT mice (Fig. 3f). Compared with those of WT mice, we subsequently analyzed the systemic metabolic characteristics of Lace1 KO mice after cold exposure and observed significant increases in both VO_2_ (Fig. 3g) and thermogenesis (Fig. 3h). Furthermore, we found that the Lace1 KO mice have higher rectal temperatures than the WT mice under cold conditions (Fig. 3i). These findings indicate that the Lace1 KO mice exhibit elevated energy expenditure when subjected to cold exposure, demonstrating an increased cold tolerance and capacity to sustain core body temperature through heightened thermogenesis. This augmented thermogenic response strongly aligns with the histological and molecular results, confirming that deficiency of the Lace1 gene promotes browning of iWAT.

### Lace1 loss promotes lactate/GPR81 signaling in CL-induced iWAT

**Fig. 4:**
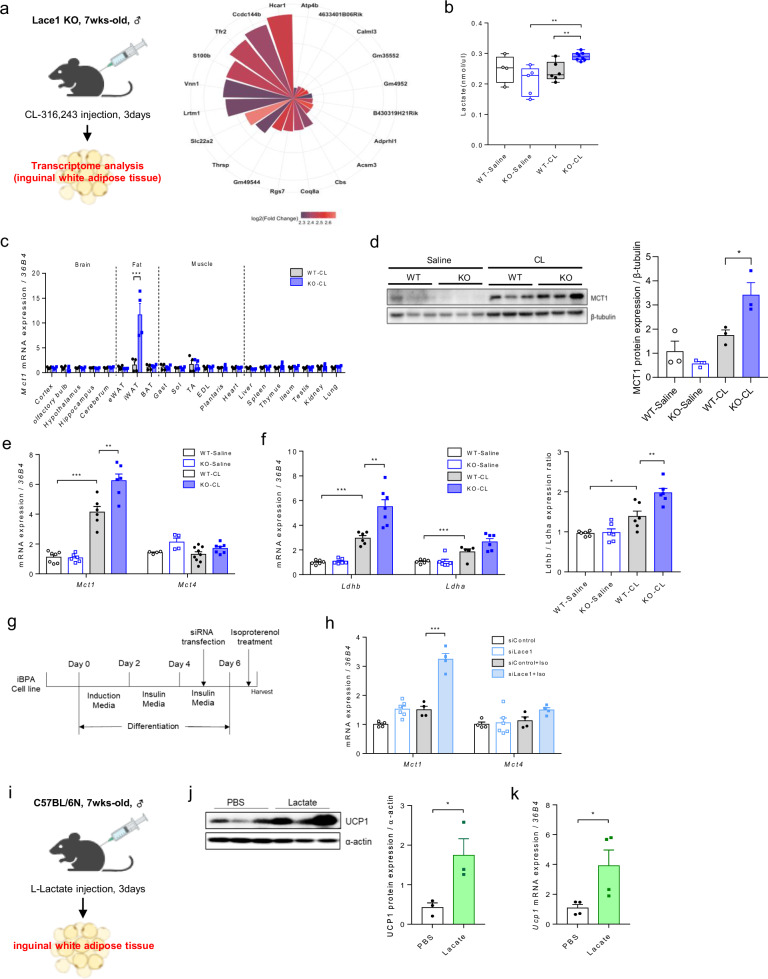
Lace1 deficiency promotes browning in CL-induced iWAT via lactate uptake and utilization. **a** Top 20 upregulated differentially expressed genes (DEGs) in iWAT from the Lace1 KO mice compared with those in iWAT from the WT mice subjected to CL challenge. **b** Circulating lactate levels in the Lace1 KO mice upon CL challenge. WT-saline; *n* = 4, KO-saline; *n* = 5, WT-CL; *n* = 6, KO-CL; *n* = 8. **c** *Mct1* mRNA levels in whole tissues from the Lace1 KO mice upon CL challenge; *n* = 4 for all groups. **d** MCT1 protein levels in the iWAT of the Lace1 KO mice subjected to CL challenge; *n* = 3 for all groups. **e** *Mct1* and *Mct4* mRNA expression in iWAT of the Lace1 KO mice under CL challenge. WT-saline; *n* = 7, KO-saline; *n* = 6, WT-CL; *n* = 6, KO-CL; *n* = 6. **f** *Ldhb* and *Ldha* mRNA expression in the iWAT of the Lace1 KO mice subjected to CL challenge; *n* = 5 for all groups. WT-saline. Ldhb/Ldha expression ratio in iWAT of the Lace1 KO mice under CL challenge. The values were calculated from the mRNA expression values; *n* = 5 for all groups. WT-saline; *n* = 6, KO-saline; *n* = 6, WT-CL; *n* = 6, KO-CL; *n* = 7. **g** Scheme of Lace1 knockdown (KD) and isoproterenol treatment of fully differentiated brown adipocytes from the iBPA cell line. **h** *Mct1* and *Mct4* mRNA expression in the Lace1-KD iBPA cells treated with isoproterenol. siControl; *n* = 4, siLace1; *n* = 6, siControl + Iso; *n* = 4, siLace1 + Iso; *n* = 4. **i** Scheme of the L-lactate challenge once a day for 3 days. **j** UCP1 protein levels after L-lactate injection; *n* = 3 for all groups. **k** *Ucp1* mRNA levels after L-lactate injection; *n* = 4 for all groups. All experiments were performed after intervention. The values are presented as the means ± SEMs. Significance was calculated via an unpaired two-tailed Student’s *t-*test. *p < 0.05, **p < 0.01, ***p < 0.001.

To investigate why Lace1 deficiency accelerates the browning of iWAT, we compared the genes whose expression was upregulated in iWAT between the WT and Lace1 KO mice subjected to CL challenge via bulk RNA sequencing (Supplementary Fig. 5). The RNA-seq data showed that hydroxycarboxylic acid receptor 1 (*Hcar1*) was upregulated in the iWAT of the Lace1 KO mice upon CL compared with that in the wild-type mice was (Fig. 4a). Hcar1 is a well-known G protein-coupled receptor gene that functions as a receptor for lactate^21,22^. Hcar1, also known as Gpr81, is expressed in adipose tissue, liver, and muscle, and its upregulation in iWAT has been reported in response to CL challenge^23^. On the basis of our sequencing results and literature, we hypothesized that the Lace1 KO mice exhibit greater browning capacity than the WT mice through lactate utilization. Lactate, a well-known promoter of browning, serves as the foundation for this hypothesis^24^. Initially, we quantified the serum L-lactate levels in response to CL treatment. Intriguingly, we observed an elevated amount of circulating L-lactate in the CL-treated Lace1 KO mice compared with the WT mice (Fig. 4b).

We proceeded to assess the expression levels of monocarboxylate transporter 1 (Mct1), which is responsible for lactate influx, to gauge the lactate uptake capacity in the iWAT of the Lace1 KO mice. First, we analyzed *Mct1* mRNA levels in the entire tissues of the CL-treated Lace1 KO mice. Notably, *Mct1* mRNA expression increased more than 10-fold exclusively in the iWAT of the Lace1 KO mice upon CL treatment, with no concurrent alterations in other tissues (Fig. 4c). Additionally, we observed a substantial increase in MCT1 protein levels, specifically in the iWAT of the Lace1 KO mice subjected to CL treatment (Fig. 4d). Conversely, the expression of the lactate efflux transporter monocarboxylate transporter 4 (Mct4) remained unaltered in the iWAT of the Lace1 KO mice subjected to CL challenge (Fig. 4e). These findings collectively indicate that, in comparison with their control counterparts, the Lace1-deficient mice exhibit increased lactate uptake while concurrently impeding lactate efflux in iWAT during CL challenge.

Lactate can be converted to pyruvate to increase Ucp1 expression via lactate dehydrogenase (LDH) in the mitochondria of iWAT and BAT^24^. LDHB functions to convert lactate to pyruvate; in contrast, LDHA converts pyruvate to lactate^25^. Hence, we proceeded to assess the mRNA expression of *Ldhb* and *Ldha* in iWAT following CL challenge. The results revealed elevated *Ldhb* mRNA expression in the Lace1 KO mice, whereas *Ldha* mRNA expression remained unchanged compared with that in the CL-treated controls (Fig. 4f). Under cold conditions for 7 days, *Mct1* mRNA levels, but not *Mct4 levels*, in the iWAT of the Lace1 KO mice showed a trend toward increased expression, as in CL conditions (Supplementary Fig. 6a). In addition, *Ldhb* but not Ldha mRNA expression was increased (Supplementary Fig. 6b and c). Collectively, these findings suggest that Lace1-deficient mice exhibit increased lactate uptake in their iWAT following CL challenge or cold exposure, with subsequent active conversion to pyruvate.

For comprehensive verification, we performed Lace1 gene knockdown in a differentiated BAT cell line, followed by the induction of adrenergic receptor activity through isoproterenol stimulation. Intriguingly, this manipulation resulted in elevated Mct1 gene expression, mirroring the in vivo observations (Fig. 4g and h). Furthermore, when L-lactate was administered once daily for 3 days to 7-week-old male C57BL/6N mice, we noted a significant increase in *Ucp1* protein and mRNA expression within the iWAT (Fig. 4i–k). L-lactate, a well-recognized metabolite produced from pyruvate during anaerobic glycolysis^26^, has gained prominence as a novel metabolic regulator, with recent studies highlighting its role in white adipose tissue browning^23,24^. Lace1 knockout in mice is thought to regulate lactate metabolism, potentially through the induction of white fat browning.

### Loss of Lace1 results in inactivation of pyruvate dehydrogenase, which promotes lactate efflux in CL-induced hearts

**Fig. 5:**
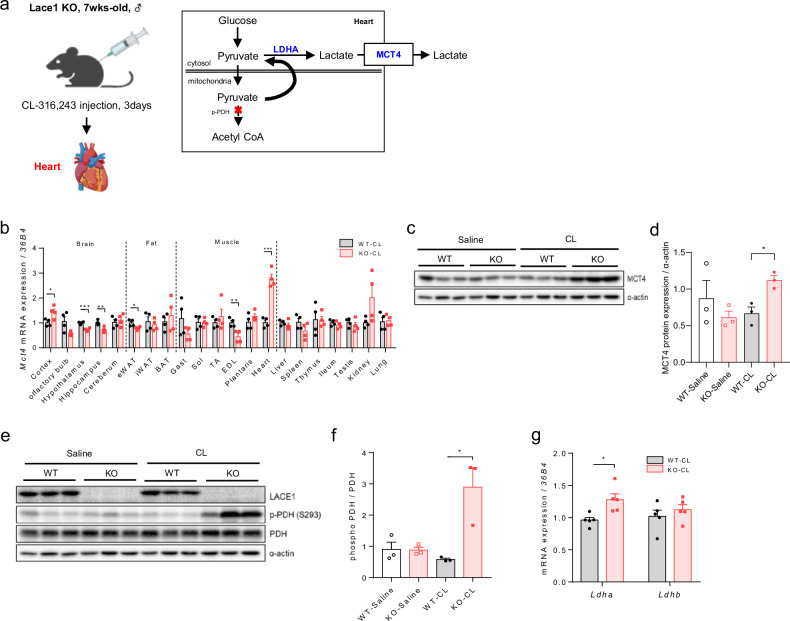
Lace1 deficiency promotes lactate efflux in CL-induced hearts. **a** Scheme of CL challenge once a day for 3 days and the mechanisms of lactate release via phosphorylation of pyruvate dehydrogenase and the mechanisms of lactate release via phosphorylation of pyruvate dehydrogenase in the heart. **b** *Mct4* mRNA levels in the whole tissue of the Lace1 KO mice upon CL challenge; *n* = 4 for all groups. **c**, **d** MCT4 protein levels in the hearts of the Lace1 KO mice subjected to CL challenge; *n* = 3 for all groups. **d**–**f** Lace1, phospho-PDH (Ser293) and total PDH protein levels in the hearts of the Lace1 KO mice subjected to CL challenge; *n* = 3 for all groups. **g** *Ldha* and *Ldhb* mRNA expression in the hearts of the Lace1 KO mice subjected to CL challenge; *n* = 5 for all groups.

**Fig. 6:**
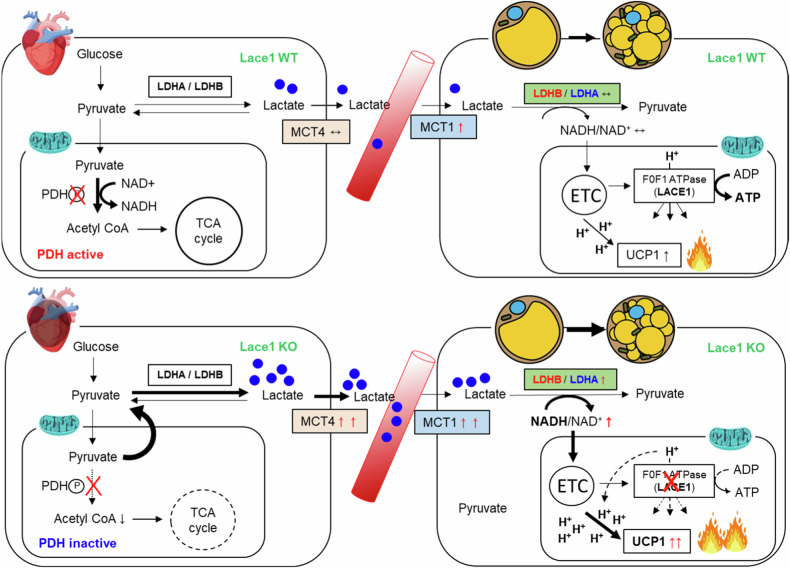
A proposed model of lactate-induced browning in Lace1 KO mice. Scheme of lactate-induced browning of iWAT in Lace1 KO mice.

To investigate why Lace1 KO mice have a high amount of circulating L-lactate upon CL challenge, we measured *Mct4* mRNA expression, which is related to a lactate efflux, in whole tissues (Fig. 5b). Lace1 KO mice presented highly increased *Mct4* mRNA and protein expression in the heart (Fig. 5b–d). However, MCT4 protein expression, which was significantly increased in the hearts of the CL-treated KO mice, did not differ in the soleus, gas, or kidney (Supplementary Fig. 7). On the basis of the *Mct4* mRNA level in the hearts of the Lace1 KO mice subjected to CL challenge, we hypothesized that lactate released from the heart contributes to lactate uptake and utilization for browning in Lace1 KO mice subjected to CL challenge. Lactate release through Mct4 activation in heart tissue is a cardiac hypertrophic phenotype^27^. Cardiac hypertrophy can progress to heart failure through increasing cardiomyocyte and heart size^27,28^. However, hypertrophic morphology was not observed in the hearts of the Lace1 KO mice subjected to CL challenge. Nevertheless, we found that the hearts of the Lace1 KO mice displayed pyruvate dehydrogenase (PDH) inactivation compared with those of the WT mice under CL challenge (Fig. 5e and f). When PDH is inactive, pyruvate from glycolysis cannot be converted to acetyl-CoA, thereby converting pyruvate to lactate. With an unbalanced pyruvate‒lactate axis, lactate is released into the blood^27^ (Fig. 5a). Additionally, *Ldha* mRNA levels were increased in the hearts of the Lace1 KO mice under CL challenge (Fig. 5g). This finding is in stark contrast to the observed increase in MCT1 and LDHB levels following CL stimulation in iWAT. Taken together, our data revealed the inactivation of PDH in the hearts of the Lace1 KO mice during CL challenge, alongside increased lactate efflux through MCT4 via increased LDHA expression. Moreover, we propose that these mechanisms may have contributed to lactate uptake-induced browning in iWAT (Fig. 6).

## Discussion

The current study examined the role of Lace1 in the browning of iWAT. Lace1 expression was substantially increased in iWAT by browning stimuli such as CL injection and cold exposure. However, the browning capacity of the Lace1-deficient mice was not disrupted; rather, the Lace1-deficient mice presented increased browning capacity. To understand these conflicting consequences, we investigated the mechanisms by which Lace1 deficiency promotes browning capacity in mice. Here, we observed that CL-induced upregulation of browning in Lace1-deficient mice was stimulated by lactate uptake in iWAT, suggesting for the first time that this phenomenon is due to increased lactate secretion induced by PDH phosphorylation in the hearts of Lace1 KO mice.

First, our data clearly revealed the expression of Lace1 in the browning process of iWAT, such as CL stimulation and cold exposure, which was significantly correlated with the Ucp1 level. Ucp1 is the most representative marker of adipose tissue browning and is predominantly expressed in cells where many mitochondria are found, such as BAT^3,29^. Thus, accumulating evidence has revealed the mechanism of Ucp1 expression in brown adipocytes and beige adipocytes with a high content of mitochondria and its effect on whole-body energy homeostasis. Therefore, we concluded that Lace1 is a gene that plays an important role in the browning process.

β3-ARs are the predominant regulators of WAT browning, and CL is a potent and highly selective β3-AR agonist^30^. According to many studies related to WAT browning, CL treatment significantly increased browning marker gene expression, reduced lipid droplet size, and increased heat generation and energy expenditure in both BAT and iWAT^31,32^, which was more evident over time. Since we identified Lace1 as an important gene for browning, we administered CL to Lace1 KO mice and investigated the importance of Lace1 in the browning process. Interestingly, the Lace1 KO mice presented smaller lipid droplets and greater Ucp1 expression following CL treatment. Furthermore, oxygen uptake capacity and heat generation were significantly greater in the KO mice than in the WT mice. This phenomenon was the same with cold exposure.

To identify the cause or mediator of these unexpected results, we performed RNA-seq to investigate which gene had altered expression in CL-induced iWAT in Lace1 KO mice. We found that the expression of the *Hcar1* gene was the highest in iWAT when the CL was administered to Lace1 KO mice, which prompted us to establish a new hypothesis in this study. Hcar1, also known as GPR81, is a cell-surface receptor for lactate and is highly expressed in adipose tissues but is also found in the kidney, skeletal muscle, and liver in mammals^23^. Lactate-induced GPR81 effectively suppresses lipolysis via modulation of the cyclic adenosine monophosphate (cAMP)-protein kinase A (PKA) pathway, which contributes to hormone-sensitive lipase (HSL) downregulation in adipose tissue^32,33^. Furthermore, the activation of the GPR81-induced cAMP‒PKA pathway is synergistically dependent on antilipolytic effects and insulin actions^34^. These results suggest that lactate can modulate metabolic processes via Gpr81-dependent mechanisms in adipose tissue.

Lactate may play a role in browning as a metabolite. Recent studies reported that lactate plays a crucial role in the browning of WAT through its receptor GPR81 and transporter Mct1 in a β3-AR stimulation-dependent manner^23,35^. On the basis of a previous study and our RNA-seq data, we hypothesized that CL-induced Hcar1 gene expression in the iWAT of the Lace1 KO mice could be due to increased circulating lactate levels. As expected, the serum lactate level was significantly greater in the CL-induced Lace1 KO mice than in the WT mice, and the *Hcar1* receptor level was significantly elevated in the Lace KO mice. In addition, we confirmed the lactate uptake ability of the Lace1 KO mice by CL stimulation and confirmed that Mct1, a lactate influx transporter, was specifically expressed only in iWAT. Lagarde et al. reported a positive correlation between Mct1 and thermogenic marker gene levels in iWAT. These researchers also revealed that Ucp1-positive cells were highly expressed in the subpopulation of adipocytes expressing Mct1 after cold exposure. Lactate is first converted to pyruvate by the LDH reaction for oxidation, indicating that lactate consumption is directly dependent on mitochondrial pyruvate utilization^26,36^. Compared with the WT mice, the CL-induced Lace1 KO mice presented significantly elevated Ldhb expression levels in iWAT. These results suggest that CL-stimulated WAT from Lace1 KO mice takes up higher levels of lactate via upregulation of Mct1 than does that from WT mice and increases Ucp1 expression by increasing oxidative phosphorylation in mitochondria through a high rate of conversion to pyruvate. We believe that these data provide essential insight into the role of lactate in β3-AR-induced browning of iWAT.

Although we found that Lace1 KO mice displayed increased browning capacity through lactate uptake upon CL stimulation, lactate concentrations did not differ in iWAT between WT and KO mice. Therefore, we aimed to determine where and why lactate increases, and we measured Mct4, a lactate exporter, in whole tissues of the CL-stimulated WT mice and KO mice via the same method as that used for Mct1 in iWAT. Interestingly, in contrast to the Mct1 results in iWAT, the CL-induced Lace1 KO mice exhibited exclusively increased Mct4 expression in the heart compared with the WT mice. Under normal conditions, cardiomyocytes express low levels of Mct4, whose levels are increased by hypertrophy or heart failure^27,28^. However, we did not observe hypertrophic morphology in the CL-induced Lace1 KO mice. Unlike previous reports, there was no difference in the cardiac hypertrophic phenotype according to elevated Mct4. Nevertheless, we noted that PDH phosphorylation was significantly increased only in the Lace1 KO mice upon CL treatment. PDH is the rate-limiting enzyme for glucose oxidation, and cardiac-specific deletion of PDH impairs glucose oxidation in mice^37^. Our results suggest that the increase in MCT4 in the CL-induced Lace1 KO mice is due to impaired pyruvate flux into mitochondria caused by PDH phosphorylation. The role of β3-ARs in the heart has long been debated. This receptor could have either a protective effect or a detrimental effect on the heart^38,39^. However, studies on the effect of CL-specific β3-AR activation on the heart have not been conducted, which is a limitation of the discussion in our study. Further studies are needed to investigate the molecular mechanisms that may explain how LACE1 KO blocks PDH in a cardiac-specific manner and the mechanisms that increase MCT4 expression. These results would ultimately provide an important basis for explaining the lactate-induced browning of iWAT observed in this study and could be the start of a new line of research to elucidate the relationship between cardiac metabolism and adipocyte browning.

Ikeda et al. suggested two reasons for the importance of beige fat studies. First, beige fat is a unique model for understanding how environmental factors control cell fate and maintenance. Second, beige fat is relevant to adult humans and may be a therapeutic target in the treatment of metabolic disorders^40^. We first suggested that Lace1 is a new gene that regulates the browning of white adipose tissue, which is the main target tissue of obesity metabolic research. Furthermore, we first suggested that CL, a specific agonist of β3-ARs, which we used as a representative tool in browning research, regulates the browning of white fat by modulating the molecular and physiological functions of the heart through the Lace1 gene. Therefore, the Lace1 gene is expected to play a role as an important therapeutic target in the future study of adipose tissue browning, obesity and metabolism.

## Supplementary information

Supplementary Figures
