# Supplementary material for: Lactate utilization in Lace1 knockout mice promotes browning of inguinal white adipose tissue
Source: Exp Mol Med. 2024 Nov 7;56(11):2491–502. doi: 10.1038/s12276-024-01324-w (PMC11612233; doi:10.1038/s12276-024-01324-w)
Supplement: Supplementary file 1 — Supplementary Figures [file 12276_2024_1324_MOESM1_ESM.pdf]

Supplementary Figure

Lactate Utilization in Lace1 Knockout Mice Promotes Browning of Inguinal White Adipose Tissue.

Kim et al.,

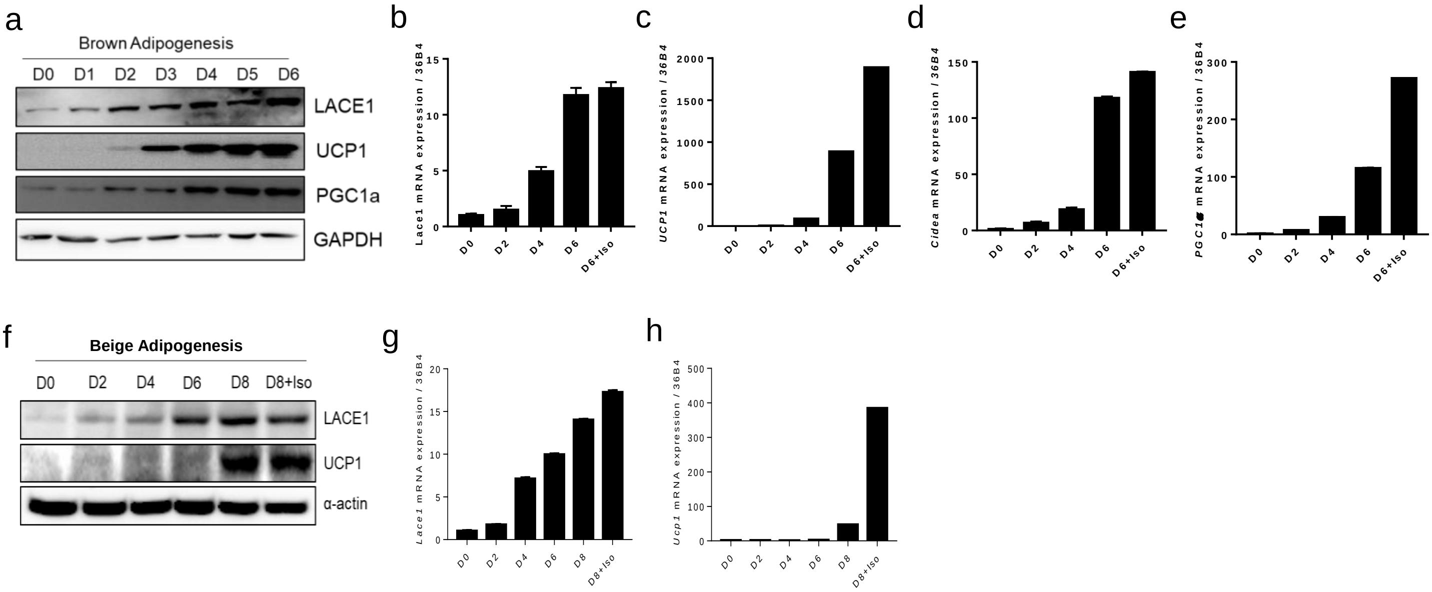

Supplementary Fig.1 The expression of Lace1 in brown adipogenesis and beige adipogenesis

(a) LACE1, UCP1 and PGC1α protein expression during brown adipogenesis. (b) *Lace1* mRNA expression during brown adipogenesis; n=3 for all group. (c) *Ucp1* mRNA expression during brown adipogenesis; n=3 for all group. (d) *Cidea* mRNA expression during brown adipogenesis; n=3 for all group. (e) *Pgc1α* mRNA expression during brown adipogenesis; n=3 for all group. (f) LACE1 and UCP1 protein expression during beige adipogenesis. (g) *Lace1* mRNA expression during beige adipogenesis; n=3 for all group. (h) *Ucp1* mRNA expression during beige adipogenesis; n=3 for all group.

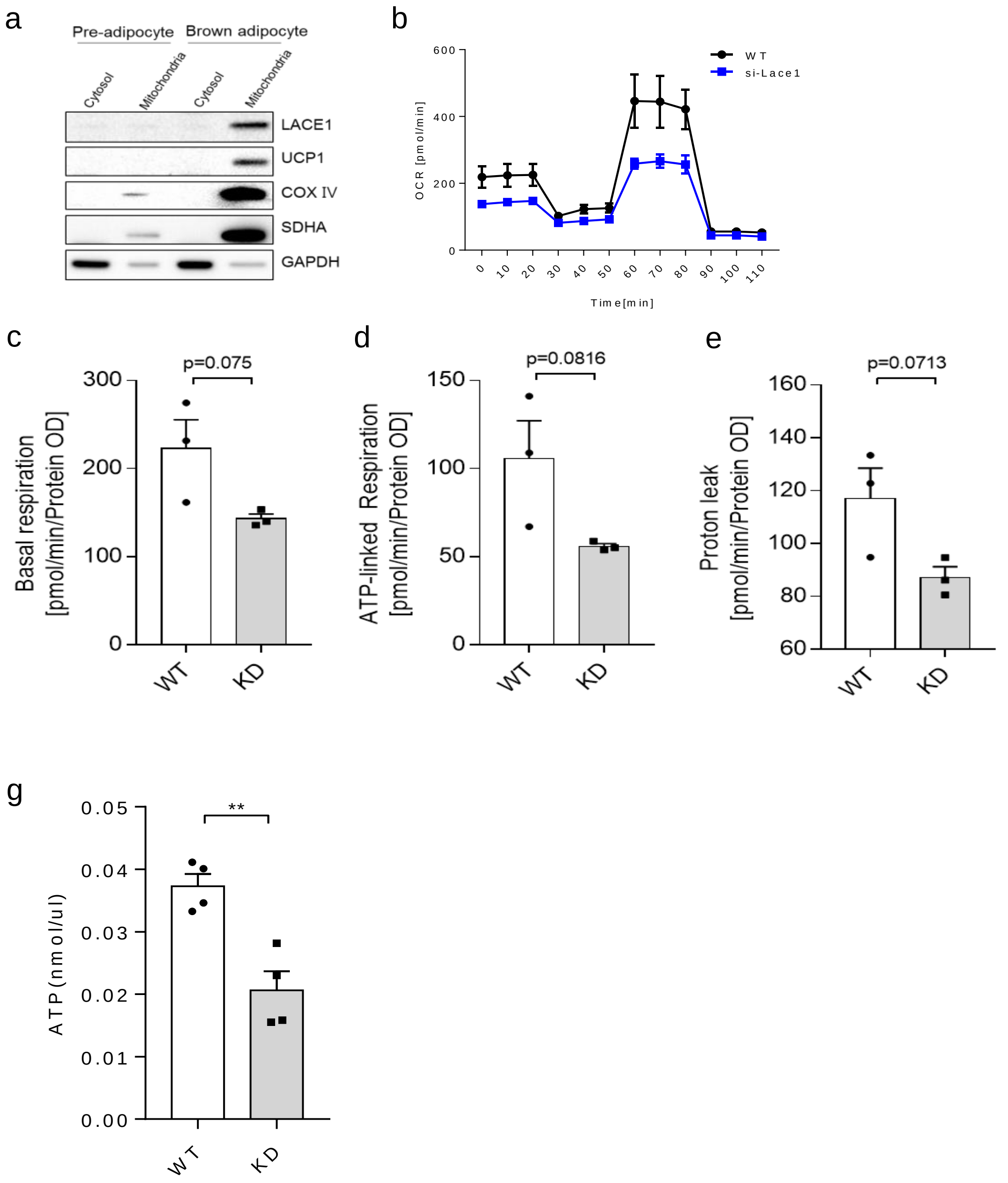

Supplementary Fig.2 Lace1 is a mitochondrial protein and ATP synthase

(a) LACE1, UCP1, COX Ⅳ, SDHA and GAPDH protein expression in cytosol and mitochondria of pre-brown adipocytes and fully-differentiated brown adipocyte using immortalized brown pre-adipocytes (iBPA) cell lines. (b-f) Oxygen consumption ratio (OCR) of Lace1 knock down (KD) in fully-differentiated brown adipocytes of iBPA cell line; n=3 for all group. (g) ATP level of Lace1 KD in fully-differentiated brown adipocytes of iBPA cell line; n=4 for all group. All experiments were performed after intervention. Values in show mean ± SEM. Significance was calculated using unpaired two tailed student's t-test. *p<0.05, **p<0.01, ***p< 0.001

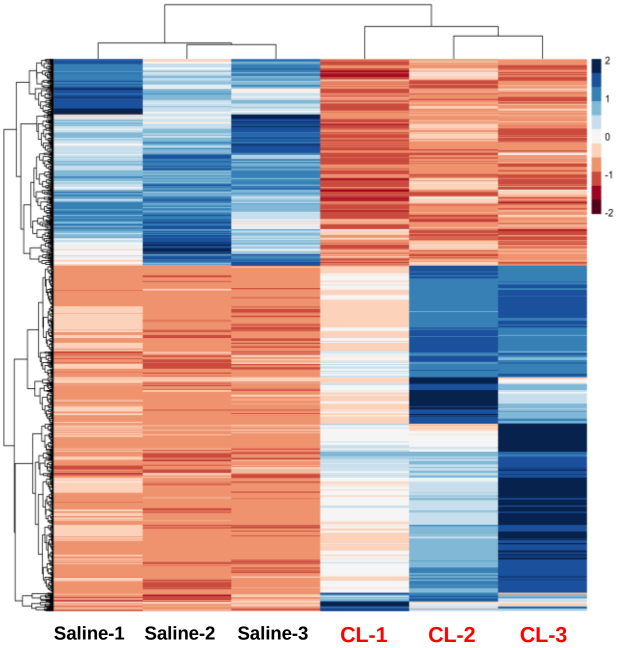

Supplementary Fig.3 Heat map of the hierarchical clustering in iWAT of C57BL/6 mice under CL-316,243 challenge

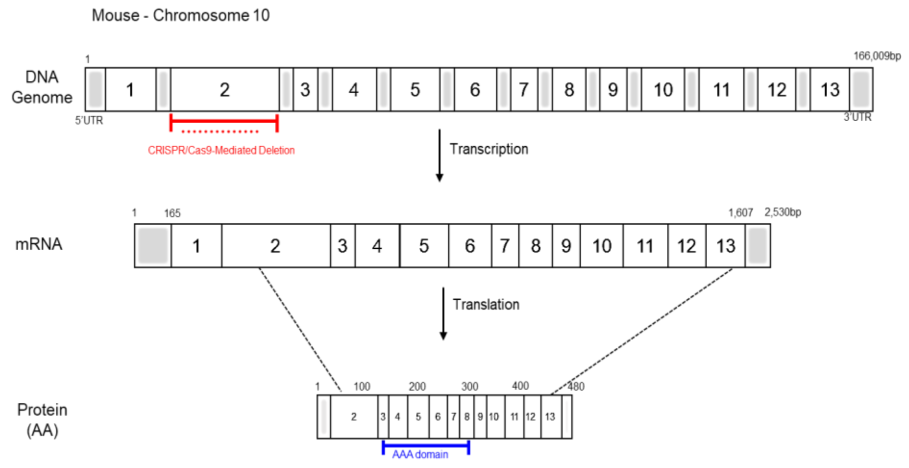

Supplementary Fig.4 A schematic of the Lace1 knockout mouse

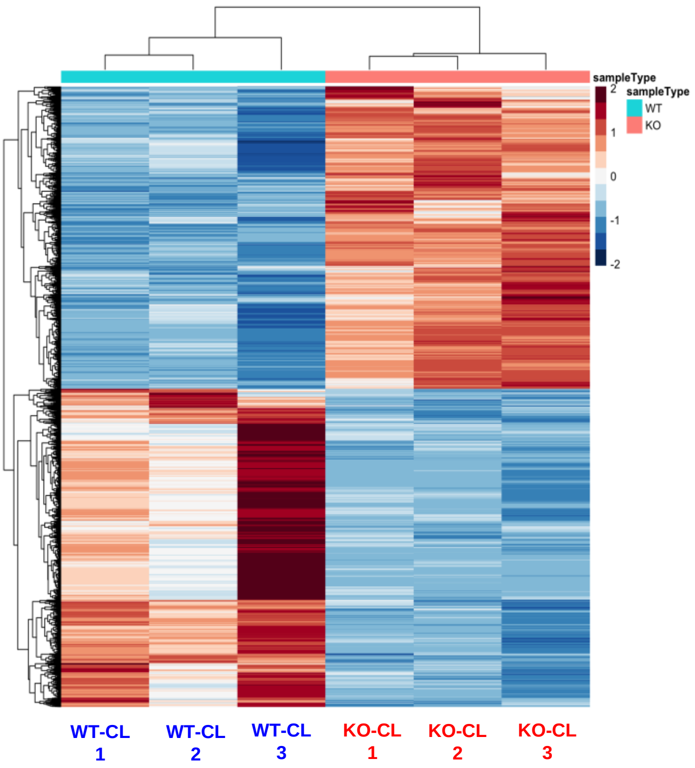

Supplementary Fig.5 Heat map of the hierarchical clustering in iWAT of Lace1 KO mice under CL-316,243 challenge

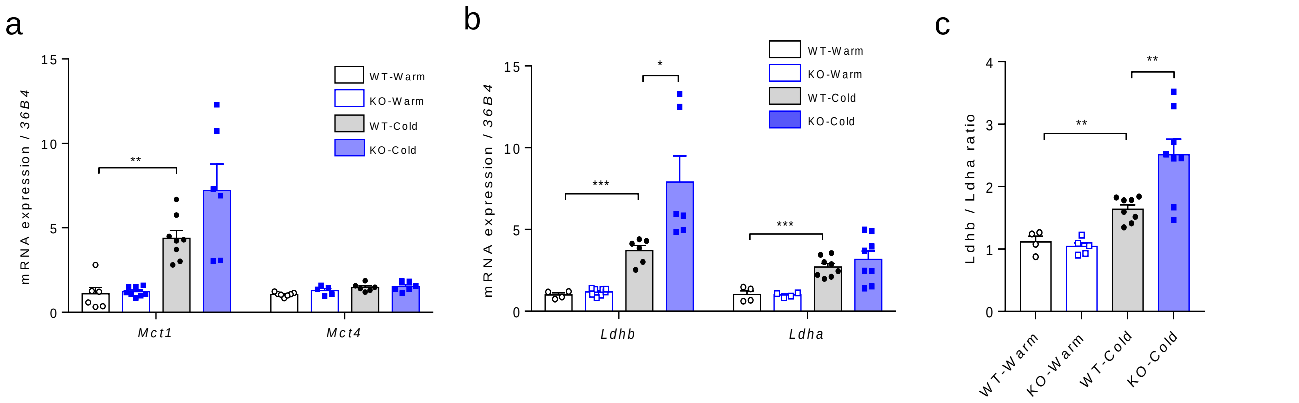

Supplementary Fig.6 Lactate influx and efflux related gene expression in iWAT upon cold exposure.

(a) *Mct1* and *Mct4* mRNA expression in iWAT of Lace1 KO mice compared to WT upon cold exposure. WT-Saline; n=6, KO-Saline; n=8, WT-CL; n=8, KO-CL; n=6. (b) *Ldhb* and *Ldha* mRNA expression in iWAT of Lace1 KO mice compared to WT upon cold exposure. WT-Saline; n=4, KO-Saline; n=8, WT-CL; n=6, KO-CL; n=6. (c) *Ldhb* / *Ldha* ratio. WT-Saline; n=4, KO-Saline; n=8, WT-CL; n=6, KO-CL; n=6. All experiments were performed after intervention. Values in show mean ± SEM. Significance was calculated using unpaired two tailed student’s t-test. *p<0.05, **p<0.01, ***p< 0.001.

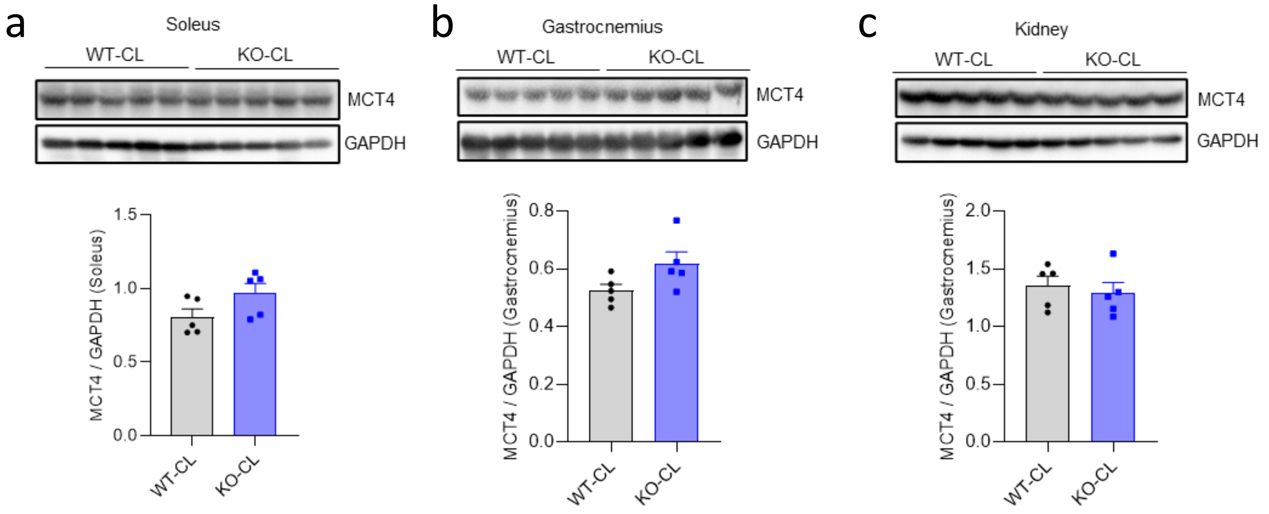

Supplementary Fig.7 Lace1 deficiency is not changed MCT4 protein level in skeletal muscle (soleus, gastrocnemius) and kidney tissues upon CL challenge.

(a) MCT4 protein expression in soleus muscle of Lace1 KO mice under CL challenge. WT-CL; n=5, KO-CL; n=5. (b) Mct4 protein expression in gastrocnemius muscle of Lace1 KO mice under CL challenge. WT-CL; n=5, KO-CL; n=5. (c) Mct4 protein expression in kidney muscle of Lace1 KO mice under CL challenge. WT-CL; n=5, KO-CL; n=5.
